# The evolution of a bat population with white-nose syndrome (WNS) reveals a shift from an epizootic to an enzootic phase

**DOI:** 10.1186/s12983-019-0340-y

**Published:** 2019-12-03

**Authors:** Craig L. Frank, April D. Davis, Carl Herzog

**Affiliations:** 1 Department of Biological Sciences, Fordham University, The Louis Calder Center, P.O. Box 887, Armonk, NY 10504 USA; 2 Griffin Laboratory, Wadsworth Center, New York State Department of Health, 5668 State Farm Road, Slingerlands, NY 12159 USA; 3 New York State Department of Environmental Conservation, 625 Broadway, Albany, NY 12233 USA

**Keywords:** White-nose syndrome, *Myotis lucifugus*, *Pseudogymnoascus destructans*, Hibernation, Torpor, Mycosis

## Abstract

**Background:**

White-nose Syndrome (WNS) is a mycosis caused by a cutaneous infection with the fungus *Pseudogymnoascus destructans* (*Pd*). It produces hibernation mortality rates of 75–98% in 4 bats: *Myotis lucifugus*, *M. septentrionalis*, *M. sodalis*, and *Perimyotis subflavus*. These high mortality rates were observed during the first several years after the arrival of *P. destructans* at a hibernation site. Mortality is caused by a 60% decrease in torpor bout duration, which results in a premature depletion of depot fat prior to spring.

**Results:**

Little is known about the long-term effects of *Pd* on torpor and mortality, thus we conducted a 9-year study on *M. lucifugus* at 5 of the hibernation sites where *Pd* first appeared in North America during the winter of 2007–08. The *M. lucifugus* hibernating at one of these sites one year after the arrival of *Pd* (2008–09) had: a) a mean torpor bout duration of 7.6 d, b) no depot fat reserves by March, and c) an apparent over-winter mortality rate of 88%. The *M. lucifugus* hibernating at this same site 6–9 years after the arrival of *Pd*, in contrast, had: a) a mean torpor bout duration of 14.7 d, b) depot fat remaining in March, and c) an apparent mortality rate of 50%. The number of *M. lucifugus* hibernating at 2 of these sites has consistently increased since 2010 and is now more than 3.0-fold higher than the number remaining after the winter of 2008–09.

**Conclusions:**

These findings indicate that this population of *M. lucifugus* has evolved mechanisms to hibernate well in the presence of *Pd*, thus reducing over-winter mortality.

## Background

White-nose Syndrome (WNS) is an emergent mycosis caused by an extensive cutaneous infection with the fungus *Pseudogymnoascus destructans* (*Pd*). It was first observed at a single cave in New York State during the winter of 2006–2007, and then spread to 5 more caves/mines in New York State during the winter of 2007–08 [1]. *Pseudogymnoascus destructans* (*Pd*) has since spread to 38 U.S. States and 7 Canadian provinces, and it was introduced to North America from Europe [2]. This fungus grows on the muzzle, wings, and ears of afflicted bats during hibernation, with hyphae penetrating both the epidermis and dermis, consuming the hair follicles, sebaceous and sweat glands [3–5]. The optimal temperature for *Pd* growth is ~ 12.5 °C and it ceases above 19 °C [6]. Within 1–2 years after the arrival of *Pd* at a hibernation site, the number of little brown (*Myotis lucifugus*), northern long-eared (*Myotis septentrionalis*), Indiana (*Myotis sodalis*), and tricolored (*Perimyotis subflavus*) bats decreases by 75–95% [7].

The body fat content of *M. lucifugus* increases from 7 to 27% body mass during the 1–2 months prior to hibernation [8, 9]. Depot fat is the primary energy source utilized during mammalian hibernation [10, 11]. Mammalian torpor involves the regulation of body temperature (T_b_) at a substantially lower level, with a new critical minimum T_b_ maintained. Hibernators do not remain torpid throughout the hibernation season; instead bouts of torpor last from days to weeks, interrupted by brief (< 3 h for bats) periods of high metabolic rates and T_b_, known as arousal episodes [12], which account for ~ 90% of the depot fat utilized during hibernation, but their physiological function is unknown [13]. Field studies on *M. lucifugus* indicate that cutaneous infection with *Pd* causes mortality through the disruption of normal torpor patterns during hibernation, causing more frequent arousal episodes, which leads to a premature depletion of depot fat reserves prior to the availability of food, and subsequent death [14].

Little is known about the long-term effects of *Pd* on bat populations, however, beyond the declines observed during the first 1–2 years. A mark/recapture study conducted by Reichard et al. [15] revealed that the over-winter survival rate of *M. lucifugus* at 8 *Pd* contaminated sites in the Northeastern USA was 5.4%, with some individuals surviving 4 consecutive winters. A small maternity colony of *M. lucifugus* in NY examined by Dobony and Johnson [16] during the summers of 2006 through 2017 demonstrated that the size of it decreased by 88% after the first appearance of *Pd*, then stabilized during 2010–14, and has been increasing since 2014. A field study conducted at a single hibernation site in NY during the winter of 2014–2015, about 6 years after *Pd* had arrived, indicated that the mean (± SE) torpor bout duration of *M. lucifugus* surviving the winter was 12.0 ± 10.8 d [17], which is close to the normal torpor bout duration of 15–20 d previously reported for this species [14, 18]. These studies indicate that *M. lucifugus* populations in the Northeastern USA are evolving a resistance to *Pd* infections. Each of these studies examined only those *M. lucifugus* that survived hibernation, however. The proportion of individuals capable of hibernating normally in the presence of *Pd*, and thus surviving the winter is not known, and it is not clear how this proportion has changed over time.

We hypothesized that the proportion of *M. lucifugus* capable hibernating normally in the *Pd-*contaminated sites of the Northeast would increase over time. We also hypothesized that the proportion *of M. lucifugus* with depot fat reserves persisting to the spring emergence from hibernation would correspondingly increase over time as well. Lastly, we also hypothesized these physiological adaptations to the presence of *Pd* lead to an increase in the size of these *M. lucifugus* populations. We tested our hypotheses in a 9-year field study on the first population of *M. lucifugus* to be affected by *Pd*. This study was conducted at 5 of the 6 hibernation sites where WNS first occurred in North America.

## Methods

### Torpor patterns of hibernating bats

The field study was conducted at the Williams Preserve Mine located in Ulster County, NY (Table 1). WNS first appeared at the Williams Preserve site during the winter of 2007–08, thus the winter of 2007–08 was thus designated as “*Pd* Season 0” for this site. Male and female bats differ slightly in their torpor patterns [19], thus the skin temperature (T_skin_) of only female *M. lucifugus* were examined. Adult *M. lucifugus* begin hibernating at the Williams Preserve Mine during late October/early November, with the subsequent emergence from hibernation occurring in late April. Fifteen adult female *M. lucifugus* were therefore collected while torpid in this mine during a single day in November/December 2008, 2013, and 2014 (45 total). Immediately upon collection, each bat was fitted with a temperature sensitive radio transmitter (Holohil models LB-2NT or LB-2XT) and released back into the mine. The transmitter was adhered to a shaved area on the back, between the shoulders (scapulas). It weighed only 0.5–0.6 g, and had a battery of life ~ 120 d. Each transmitter was previously calibrated by holding it at each of 6 sequential temperatures ranging from 0.3 to 40.0 °C in a circulating water bath and recording the rate of radio pulses emitted. These data were then used to calculate calibration equations for each transmitter individually.

**Table 1:** Hibernation sites and numbers of *Myotis lucifugus* in them during the most recent survey conducted prior to the first appearance of *Pseudogymnoascus destructans*

Site Name	Location	*Myotis lucifugus*	Survey Year
Hailes Cave	42.67°N, 74.08°W	15,374	2005
Howe Cave	43.30°N, 74.48°W	1213	2005
Schoharie Cavern	42.69°N, 74.24°W	314	1984
South Bethlehem Cave	42.52°N, 73.85°W	100	2005
Williams Preserve Mine	41.88°N, 74.10°W	87,401	1999

The skin temperature (T_skin_) of each bat was continuously measured and recorded at 10–15 min intervals by constantly monitoring radio signals using a computerized radio receiving system (ATS model 4500 s). Skin temperature (T_skin_) is equivalent to T_b_ in small bats [20]. Torpor was defined as when T_skin_ < 24 °C. Four antennas were placed inside the mine at areas where *M. lucifugus* roost during hibernation. The antennas were connected to 50 Ω cables that ran to the mine entrance and interfaced with the radio receiving system. This permitted weekly data downloads without re-entering the mine and disturbing hibernating bats. We did not re-enter the mine after the single day of capture and transmitter attachment during 2014 and 2015 until the following April, to avoid disturbing the bats while T_skin_ was being recorded. Severe WNS was observed during the winter of 2008–09, and all telemetered bats either disappeared or died by January 2009. Fifteen additional female *M. lucifugus* were thus collected while torpid on 3 February 2009 (Julian date 34), fitted with transmitters, and released back into the mine to obtain additional T_skin_ data for February 2009 and March 2009. We did not re-enter the mine again until April 2009. Radio signals were monitored and recorded throughout the following March during each of the 3 study periods.

Each of the 15 *M. lucifugus* captured during 2013 and 2014 were also screened for the wing lesions indicative of *Pd* infections using ultraviolet (UV) light prior to transmitter attachment following the techniques of Turner et al. [21]. All bats were kept in the mine, with both wings extended, while being transilluminated by a portable long-wave (365–385 nm) UV light. This technique causes an orange-yellow fluorescence of the lesions due to *Pd* infection. The presence or absence of an orange-yellow fluorescence was recorded for each wing. Groups of *M. lucifugus* were observed hibernating at numerous roost sites throughout the Williams Preserve Mine. We measured the ambient air temperature (T_a_) at a single *M. lucifugus* roost site located near the entrance during the 2008–09 (*Pd* season 1) and 2014–2015 (*Pd* season 7) periods by placing an Onset model “Tidbit v2” at this site. It was programmed to record temperatures at 1 h intervals from 1 December through 30 March. These loggers were factory calibrated before deployment, and their calibrations were verified after deployment by placing them at 0 and 30 °C in a circulating water bath.

A previous study on free-ranging *M. lucifugus* conducted before the arrival of *Pd* in North America revealed that their torpor bouts were 15–20 d in length [18], and a field study on *M. lucifugus* performed by Reeder et al. [14] revealed that *M. lucifugus* hibernating in areas of the Northeastern USA where *Pd* had not yet arrived had torpor bouts that averaged 16.33 ± 6.7 d in length. The mean torpor lengths observed for each group of bats were thus statistically compared to 16.33 d to determine if they deviated from the healthy torpor bout length previously reported for this species. The proportion of bats with torpor bouts that were > 15 d in length was also calculated for each study period since *M. lucifugus* must have torpor bouts that average at least 15 d long in order to survive the hibernation period [18]. Handling torpid bats caused them to arouse, and they re-entered torpor within 30 min upon their subsequent release. The first torpor bout observed for each bat was therefore not included in the analyses of torpor patterns, to avoid any biases due to artifacts stemming from the preceding “provoked” arousal episode. Only T_skin_ data collected after the first spontaneous (undisturbed) arousal episode recorded from each bat were included in the torpor pattern analyses. In most instances, T_skin_ data were obtained for several torpor bouts by the same bat. In these cases, the most recent torpor bout observed for that individual was included in the analysis.

### Body fat levels of free-ranging bats

Four adult *M. lucifugus* were collected 12 December 2013 (*Pd* Season 6), 4 on 13 November 2015 (*Pd* season 8), and 8 more on 16 March 2017 (*Pd* Season 9). All were collected while torpid from the Williams Preserve Mine, sacrificed immediately upon capture using an Isoflurane overdose, and their carcasses were frozen at − 20 °C until analysis. Adult *M. lucifugus* are sometimes infected with rabies [22] which can also lead to rapid body fat loss during hibernation, thus all bat carcasses were first tested for rabies following the methods summarized in Blanton et al. [23]. None tested positive for rabies; thus all were included in the analyses. Each carcass was first weighed to the nearest 0.0001 g and dried at 60 °C for 24 h. The dried carcass was then weighed again, homogenized, and dried again at 60 °C for another 24 h. All lipids were subsequently extracted from the homogenized carcass in a Soxhlet apparatus for 12 h using petroleum ether following the techniques of Dobush et al. [24] to determine total lipid content. Chemical extraction methods remove both depot and structural lipids. The structural lipids in mammalian tissues are phospholipids, cholesterol, ceramides, wax esters, and sphingolipids, all of which are neither stored nor mobilized as energy sources. Mammalian depot lipids, in contrast, are triacylglycerols stored in adipocytes, and are both mobilized and catabolized during fasting [25]. Total body fat content (% live mass) thus represents the sum of both depot lipids, which can be mobilized to support metabolism during fasting, and structural lipids, which cannot. Mammals that have depleted all their depot lipid reserves will consequently still have total body fat contents of 4–8% live mass [26], composed entirely of structural lipids. The mean total body fat contents obtained were thus compared to the minimum body fat content reported for free-ranging *M. lucifugus*, which is 6.7% body mass [9].

Previous field studies on the torpor patterns of WNS-affected *M. lucifugus* revealed that cutaneous infections with *Pd* vary in degree of severity within the same hibernating population. Those *M. lucifugus* with the most severe *Pd* infections have torpor bouts that are 50–60% shorter in length than normal, and usually die well before the end of hibernation (April in NY). The *M. lucifugus* with moderate *Pd* infections, however*,* usually have torpor bouts that are normal in length, and typically survive to the end of hibernation [14]. We thus also compared the body fat contents obtained to those previously reported for *M. lucifugus* collected at 2 different WNS-affected hibernation sites during 2 different periods: mid-hibernation (February 2008/09) and natural emergence from hibernation (April 2008), to assess the body fat levels associated with these 2 degrees of cutaneous infection in *M. lucifugus* shortly after *Pd* arrived. They were collected from the Williams Preserve Mine and another mine located in Essex County NY [27].

### Bat surveys

The 5 sites surveyed for bats, and the number of *M. lucifugus* hibernating in each during the most recent survey conducted prior to the arrival of *Pd* in New York, are listed in Table 1. The total number of bats hibernating in each of these sites was determined at 1 to 2-year intervals starting in 2007 by visually counting all bats roosting in them during a single day-long survey each winter. Surveys were conducted by wildlife biologists from the NY State Department of Environmental Conservation (DEC) during the February–March periods of 2008–2017. WNS was first observed at Howe Cave during the winter of 2006–07, whereas WNS was first detected at all other sites listed in Table 1 during the winter of 2007–08. *Myotis lucifugus* was the most common bat at all sites both before and after the arrival of *Pd*. These 5 hibernation sites were selected because they are among the 6 sites were WNS was first observed in North America.

### Statistical analyses

Mean body fat contents, T_skin_ and torpor bout lengths were compared among groups using a one-way ANOVA (General Linear Models) procedure in conjunction with Tukey’s Highly Significant Difference (HSD) Test. Mean torpor bout lengths and body fat contents were compared previously published values using an one-tailed Student’s *t*-test for a hypothesis concerning the mean. The number of *M. lucifugus* hibernation at a study site during each survey was analyzed by Least Squares Linear Regression. All statistical methods were performed using SYSTAT version 13.2 software. The variance about each calculated mean is reported as ±1 standard error (SE).

## Results

### Torpor patterns of hibernating bats

**Fig. 1:**
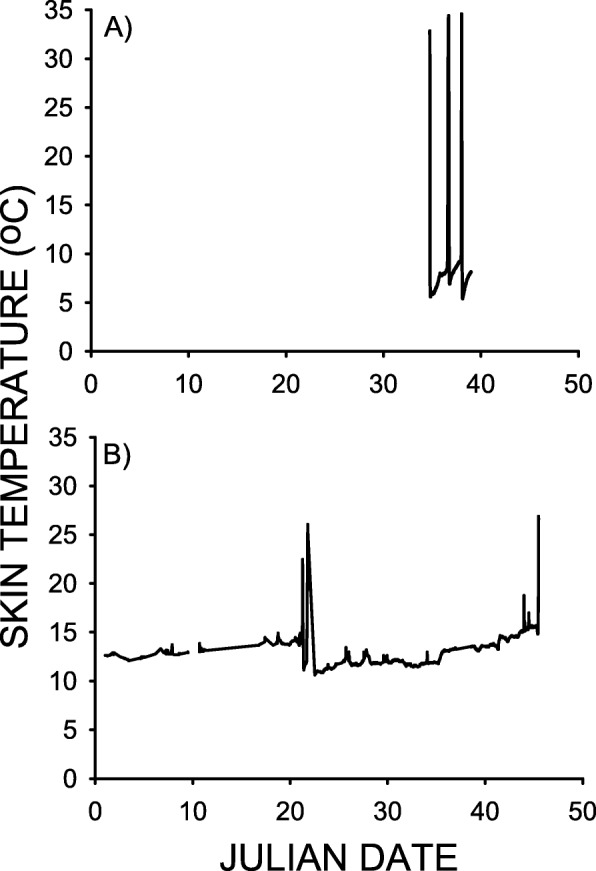
Skin temperatures of two different *Myotis lucifugus* hibernating at the Williams Preserve Mine during: **a** February 2009, and **b** January–February 2014. The February 2009 recordings of T_skin_ began 45 min after transmitters were placed on all bats on Julian date 34. Arousal episodes occurred on Julian dates 36.6458 and 38.0181, delineating a torpor bout of 1.37 d in duration. No radio signals were received from this bat after Julian date 39

Sufficient T_skin_ data were obtained to determine the spontaneous torpor patterns of 17 adult female *M. lucifugus* at the Williams Preserve Mine during the November–March period of 2008–09 (*Pd* Season 1), and for 20 more individuals during the November/December–March periods of 2013–14 and 2014–15 (*Pd* Seasons 6 and 7). The T_skin_ values recorded for a single *M. lucifugus* during February 2009, as well as for another individual observed during January and February 2014, are summarized in Fig. 1. The mean torpor bout durations observed during *Pd* Seasons 6 and 7 (Table 2) were 1.7X and 1.9X greater than that observed during *Pd* Season 1, respectively (F_2,34_ = 6.276, *p* = 0.005). Mean torpor bout duration during *Pd* Season 1 was significantly less than 16.33 d (t = − 6.291, df = 16, *p* < 0.001), but those of *Pd* Seasons 6 and 7 were not significantly different from 16.33 d (t = − 1.019, df = 12, *p* = 0.16, and, t = − 1.714, df = 6, *p* = 0.07, respectively). Just 18% of the *M. lucifugus* examined during *Pd* Season 1 (3 of 17) had torpor bouts that were > 15 d in length, whereas 50% (10 of 20 total) of the bats monitored during *Pd* seasons 6 and 7 had torpor bouts that were > 15 d. Torpor bout length varied between individual bats by more than 12-fold during *Pd* Season 1, by about 3.5-fold during *Pd* Season 6, and by just 2.6-fold during *Pd* Season 7 (Table 2). The mean minimum T_skin_ maintained during torpor in *Pd* Seasons 6 and 7 was 1.0 and 2.1 °C greater (Table 2) than that during *Pd* Season 1 (F_2,34_ = 4.178, *p* = 0.024). The mean maximum T_skin_ observed during arousal episodes was significantly less during *Pd* Season 6 (Table 2) than during *Pd* Seasons 1 and 7 (F_2,34_ = 13.267, *p* < 0.001). No visible signs of *Pd* infection were detected using UV transillumination in any of the bats captured during 2013 and 2014. Mean (± SE) December–March air temperatures at a single roost site in the mine during 2008–09 was 3.7 ± 0.1 °C, with temperatures range from 2.6 to 5.7 °C, and during this same period in 2014–15 it was 2.0 ± 0.1 °C, with temperatures ranging from − 1.0 to 4.6 °C.

**Table 2:** Mean (± SE) torpor parameters of *Myotis lucifugus* hibernating at the Williams Preserve Mine

Hibernation Period	*N*	Mean Torpor Bout (d)	Torpor Bout Range (d)	Minimum T_skin_ (°C)	Maximum T_skin_ (°C)
2008–09	17	7.58 ± 1.39^a^*	1.51–18.63	7.0 ± 0.5^a^*	33.8 ± 0.6^a^*
2013–14	13	14.68 ± 1.58^b^	6.89–24.40	8.0 ± 0.3^b^	30.0 ± 0.8^b^
2014–15	7	12.90 ± 2.00^b^	9.19–24.20	9.1 ± 0.7^b^	33.1 ± 1.0^a^

* Means within a category sharing a common lower-case letter are not statistically different at the *p* < 0.05 level

### Body fat levels of free-ranging bats

**Fig. 2:**
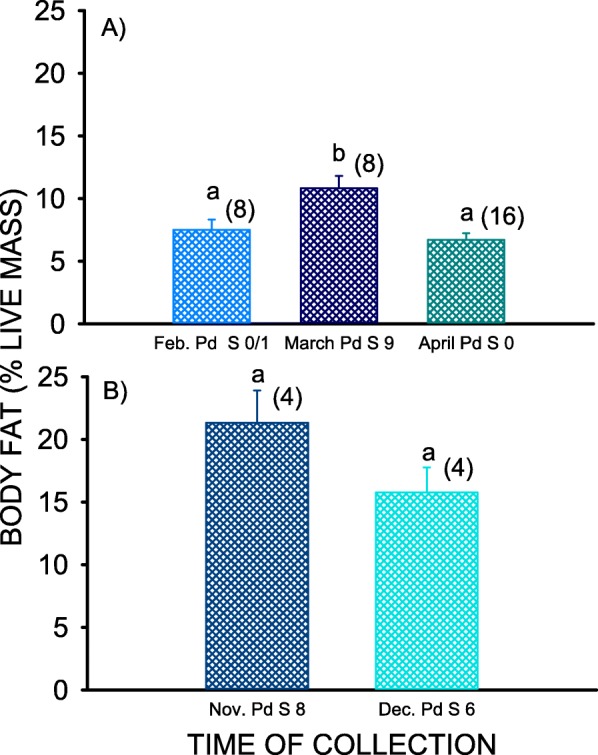
Histograms summarizing the mean (± SE) body fat contents of free-ranging *Myotis lucifugus* during **a** February–April, and **b** November–December. Mean sharing a common lower-case letter within a date range are not significantly different at the *p* < 0.05 level. Sample sizes are indicated in parentheses. Data for February 2008/09 (*Pd* Seasons 0/1) and April 2008 (*Pd* Season 0) are from Frank et al. [28]

The mean body fat content of *M. lucifugus* collected during *Pd* Season 9 (March 2017) was 1.4x greater (Fig. 2a) than that previously reported for *M. lucifugus* collected during *Pd* Seasons 0 and 1 (February 2008/09) and *Pd* Season 0 (April 2008) at mines in New York (F_2,29_ = 8.365, *p* = 0.001). The mean body fat content during February of *Pd* Seasons 0 and 1 did not significantly differ from the minimum of 6.7% body mass for *M. lucifugus* (t = 0.972, df = 7, *p* = 0.36), and neither did the April mean body fat content for *Pd* Season 0 (t = 0.045, df = 15, *p* = 0.97). The mean body fat content during the March of *Pd* Season 9, however, was significantly greater than 6.7% (t = 4.202,df = 7, *p* = 0.004). The mean body fat levels of bats collected during November and December of *Pd* Seasons 6 and 8 were not significantly different from each other (t = − 5.562, df = 6, *p* = 0.14), but when analyzed to together, the overall mean of 18.53 ± 1.84% is significantly less than the maximum of 27% body mass previously reported for this species (t = − 4.585, df = 7, *p* = 0.003). The mean (± SE) body mass of the bats collected during the November of *Pd* Season 1 was 8.6 ± 0.2 g, whereas that of the bats collected during the November/December of *Pd* Seasons 6 and 7 was 8.4 ± 0.1 g, and not significantly different (t = 0.497, df = 43, *p* = 0.62).

### Bat surveys

**Fig. 3:**
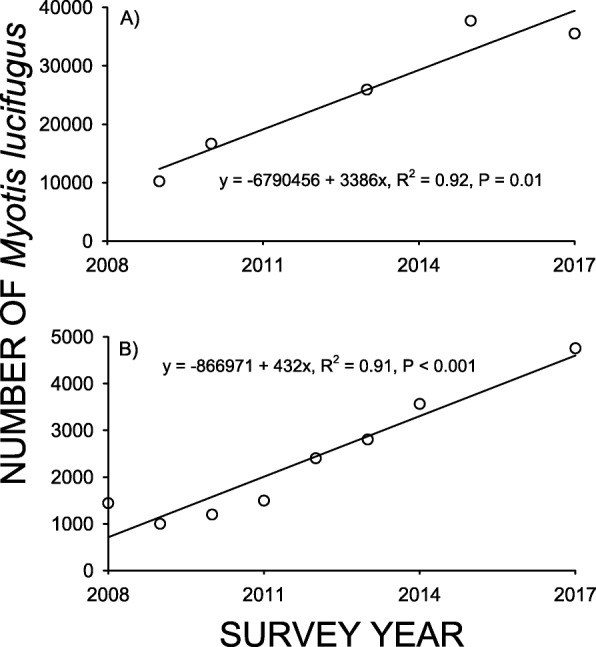
The relationship between survey year and the number of *Myotis lucifugus* hibernating at **a** The Williams Preserve Mine, and **b** Hailes Cave. The line drawn through each set of points represents the least squares linear regression

**Fig. 4:**
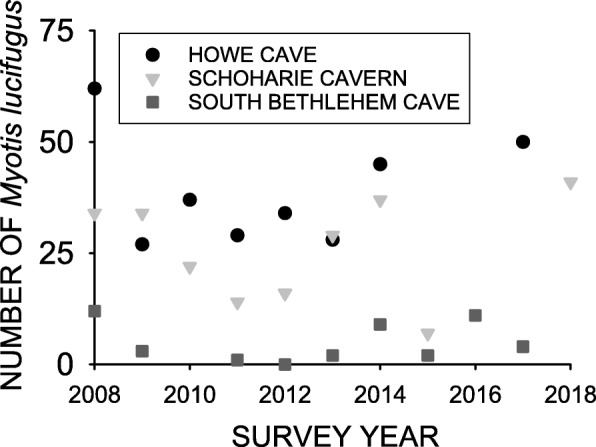
Survey year and the number of *Myotis lucifugus* hibernating at Howe Cave, Schoharie Cavern, and South Bethlehem Cave from 2008 through 2017

The number of *M. lucifugus* hibernating at the Williams Preserve Mine (Fig. 3a) during the year following the first appearance of *Pd* was just 12% of that observed at this site previously (Table 1), and the number hibernating at Hailes Cave (Fig. 3b) was just 9% of the pre-*Pd* level (Table 1). The number of *M. lucifugus* observed at these sites during subsequent surveys consistently increased, however, and the trend at each site was significant (F_1,3_ = 33.423, *p* = 0.01 for Williams Preserve Mine, and F_1,6_ = 62.986, *p* = 0.0002 for Hailes Cave). The number of *M. lucifugus* increased to 41% of the pre-*Pd* level by 2017 at the Williams Preserve Mine, and to 31% of the pre-*Pd* level for Hailes Cave by 2017 (Fig. 3). Likewise, the number of bats hibernating at Howe Cave, Schoharie Cavern, and South Bethlehem Cave were just 5–12% of the pre-*Pd* levels for these sites 1–2 years after the first appearance of *Pd* (Fig. 4). The number of *M. lucifugus* found at each these sites during subsequent survey years varied little from the initial reduction, and no significant trends were found for either Howe Cave (F_1,7_ = 0.0388, *p* = 0.84), Schoharie Cavern (F_1,6_ = 0.0184, *p* = 0.90), or South Bethlehem Cave (F_1,7_ = 0.0028, *p* = 0.96) over time.

## Discussion

The results of the field study clearly support our hypothesis that the proportion of *M. lucifugus* hibernating with normal (15–20 d) torpor bout lengths in the presence of *Pd* increases over time. Only 18% of the *M. lucifugus* observed during *Pd* Season 1 had torpor bouts that were greater than 15 d in length, whereas 50% of those observed during *Pd* Seasons 6 and 7 had torpor bouts of this duration. Consequently, mean torpor bout durations during *Pd* Seasons 6 and 7 increased by 1.7 to 1.9-fold over those that were observed during *Pd* Season 1. This increase in mean torpor bout duration was associated with a reduction in the range of torpor bout lengths observed between individuals. The shortest torpor bout observed during *Pd* Season 1 was 1.51 d, whereas the shortest torpor bout observed during *Pd* Season 7 was 9.19 d (Table 2). The increase in mean torpor bout duration was associated with a 1.0–2.1 °C increase in mean minimum T_skin_ during torpor (Table 2). This observation in in contrast to the findings of Lilley et al. [17], who observed that the T_skin_ of *M. lucifugus* hibernating at a mine where *Pd* had just arrived was greater than that of *M. lucifugus* hibernating at another mine where *Pd* had been present for about 6 years. The two study sites examined by Lilley et al. [17] differed in mean T_a_ by 4.6 °C, however, which may account for the differences in T_skin_ observed during torpor. Our study also revealed that the maximum T_skin_ observed during arousal episodes did not vary in a consistent manner between hibernation seasons, and this was also observed by Lilley et al. [17].

The air temperatures measured at a single roost site near the mine entrance range from 2.6 to 5.7 °C during the December 2008–March 2009 period, and they ranged from − 1.0 to 4.6 °C at this same site from December 2014 through March 2015. The mean minimum T_skin_ of torpid *M. lucifugus* was 7.0 ± 0.5 °C during 2008–09, and it was 9.1 ± 0.7 °C during 2014–15 (Table 2), however. The T_skin_ of *M. lucifugus* during torpor is usually 0.5–1.0 °C above ambient temperature [18], and the T_skin_ of torpid *M. lucifugus* infected with *Pd* is not significantly different from that of torpid uninfected *M. lucifugus* hibernating at the same T_a_ [28]. Field studies conducted at an abandoned mine in western Ohio (USA) revealed that *M. lucifugus* hibernate at sites with mean (± SD) surface temperatures ranging from 4.3 ± 1.6 to 10.8 ± 1.6 °C within the same mine, and that both air as well as surface temperatures are higher in the back of the mine than in the front of it, near the entrance [29, 30]. Interpreting our finding in conjunction with these published studies indicates that most of the *M. lucifugus* observed in our study were hibernating at roost sites which were further back in the Williams Preserve Mine, where it was warmer, than the single roost site at which we measured air temperatures.

The results of the body fat analyses support our hypothesis that the increase in torpor bout length associated with long-term *Pd* exposure produces a corresponding increase in the proportion of individuals with depot fat reserves lasting throughout hibernation. The mean body fat content of *M. lucifugus* hibernating during *Pd* Seasons 0–1 was not significantly different from the minimum of 6.7%, indicating that all depot fat reserves have been depleted before the end of hibernation (mid-April). The mean body fat content of *M. lucifugus* collected one month prior to end of hibernation during *Pd* Season 9 (March 2017), however, was 1.4X greater, indicating that they had some depot fat reaming at this point. The increase in the amount of depot fat was not due to an increase in the amount of depot fat at the onset of hibernation since; a) the body fat content of *M. lucifugus* during November 2015 was not significantly different from the maximum of 27% previously report for this species, and, b) the mean body masses of *M. lucifugus* collected during November 2008 and November–December 2013–15 were not significantly different. Our findings are also consistent with those of Cheng et al. [31] who observe that the *M. lucifugus* hibernating at these sites during the winter of 2009–10 had a lower mean body fat content than those hibernating at the same sites during the winter of 2015–16.

The winter counts reveal that this *M. lucifugus* population is growing. The number of individuals hibernating at all five study sites decreased to 5–12% of previous levels within several years after the arrival of *Pd*. This trend did not continue in subsequent years. The number *M. lucifugus* hibernating at the Howe Cave, Schoharie Cavern, and South Bethlehem Cave sites have since remained stable, with no significant negative, or positive, trends. The number of *M. lucifugus* hibernating at the Williams Preserve Mine and Hailes Cave sites, in contrast, significantly increased by 3 to 4-fold since the initial decline associated with the arrival of *Pd*. A field study conducted by Reeder et al. [14] on *M. lucifugus* hibernating in Northeastern USA during the first several years after the arrival of *Pd* revealed 3 categories: a) those that developed severe *Pd* infections with 7–8 d torpor bouts, all which died during hibernation, b) those that developed moderate *Pd* infections, had torpor bouts 15–20 d long, and usually survived hibernation, and, c) bats that developed no *Pd* infections, had torpor bouts of 15–20 d as well, and survived hibernation. Analyses of the amount of *Pd* DNA found on the wings of *M. lucifugus* hibernating at 4 NY study sites conducted by Langwig et al. [32] indicated that this population is developing a resistant to cutaneous infection with *Pd*. Interpreting these findings with the results of our study indicates that the proportion of *M. lucifugus* that develop only moderate, or no, *Pd* infections during hibernation is increasing in our study population.

It thus appears that since the epizootic phase of *Pd*-induced White-nose Syndrome began in the Northeastern USA, there has been a shift to an enzootic phase with respect to *M. lucifugus*, where the host and *Pd* coexist. This is similar to what has been observed for chytridiomycosis in frogs, which is caused by a cutaneous infection with the fungus *Bactrachochytrium dendrobatidis* [33]. This led to population declines for 43 of the 238 amphibian species found in Australia since 1978. About 26% of the species that initially declined are now recovering, and the population levels of an additional 19% are now stable [34]. Laboratory experiments involving two frog species that are now recovering from chytridiomycosis in Panama revealed that this recovery was not caused by pathogen attenuation but was instead due to an increase in the resistance of these frog species to cutaneous infection with *B. dendrobatidis* [35]. These studies suggest a similar scenario for the evolution of *Myotis lucifugus* populations which are resistant to *Pd* may be occurring.

It thus appears that natural selection has favored the *M. lucifugus* that can somehow limit the degree of cutaneous infection with *Pd* during hibernation to the point where torpor bout length is not significantly reduced. The proportion of *M. lucifugus* with this *Pd*-resistant phenotype in the population has apparently increased over time due to this selective advantage, producing the trends in mean torpor bout length, mean depot fat levels, and population increases observed. We hypothesize that these trends may not be due to the attenuation of the *Pd* strain found in this region. At present, only 50% of the *M. lucifugus* in this population can hibernate with torpor bouts that are > 15 d in length and survive the winter. It is unknown if proportion of individuals in this population capable of hibernating in this manner in the presence of *Pd* will continue to increase. The mechanism by which some *M. lucifugus* reduce the level of *Pd* infection is not known. Recent studies on the epidermal lipid mixtures of 2 bat species that are highly resistant to *Pd* growth reveal that some of the free fatty acids, wax esters, and monoacylglycerols found in their epidermis inhibit *Pd* growth by 90–99% [36, 37]. Studies on free-ranging *M. lucifugus* have also revealed that the proportion of anti-*Pd* fatty acids in their epidermis greatly decreases during hibernation [38]. It is thus possible that the *M. lucifugus* that are more resistant to *Pd* growth have greater levels of anti*-Pd* free fatty acids, wax esters, and/or monoacylglycerols in their epidermis during hibernation than those that are highly susceptible to these infections. Further examination of this system will not only provide important insights into the log-term effects of White-nose Syndrome, but also will illuminate how epidemics of fungal pathogens subside in general.

## Conclusions

One goal of this study was to examine the extent to which a hibernating bat population has evolved a resistance to a novel fungal pathogen (*Pd*) after 6–9 years of exposure to it. Another goal was to elucidate the physiological basis of any increase in resistance observed during this period. Our study clearly demonstrates that over a 6-year period, over-winter mortality decreased from an initial 88 to 50%. This decrease in mortality was associated with a nearly 2-fold increase in mean torpor bout length during hibernation, which in turn produced a corresponding increase in depot fat reserves during late hibernation. These findings indicate that instead of increasing the amount of body fat stored at the onset of hibernation, this bat population instead evolved mechanisms which increased the ability to maintain proper torpor patterns in the presence of *Pd*, thereby conserving depot fat reserves. This study thus demonstrates that a mammalian species is capable of rapid adaptive evolution in response to a novel fungal pathogen.

## Data Availability

The datasets supporting the conclusions of this article can be found at the Figshare web site: https://figshare.com/

## Abbreviations

df: Degree of freedom
N: Sample size
*Pd*: *Pseudogymnoascus destructans*
SD: Standard deviation
SE: Standard error
T_a_: Ambient (air) temperature
T_b_: Body temperature
T_skin_: Skin temperature
